# A drone imagery dataset for semantic segmentation of urban garden ground covers in biodiversity studies

**DOI:** 10.1038/s41597-026-07152-z

**Published:** 2026-04-08

**Authors:** Yasamin Afrasiabian, Chenghao Lu, Anirudh Belwalkar, Hany Elsharawy, Xiaoxin Song, Ying Yuan, Fei Wu, Xiang Su, Elisa Van Cleemput, Monika Egerer, Kang Yu

**Affiliations:** 1 Precision Agriculture Lab, TUM School of Life Sciences, Technical University of Munich, Dürnast 9, 85354 Freising, Germany; 2 Department of Agricultural Sciences, University of Helsinki, Helsinki, Finland; 3 Department of Computer Science, University of Helsinki, Helsinki, Finland; 4 Leiden University College The Hague, Leiden University, Anna van Buerenplein 301, 2595 DG Den Haag, The Netherlands; 5 Urban Productive Ecosystems, TUM School of Life Sciences, Technical University of Munich, Hans Carl-von-Carlowitz-Platz 2, 85354 Freising, Germany

**Keywords:** Urban ecology, Biodiversity

## Abstract

Urban gardens promote urban biodiversity by providing diverse ground covers that support habitat provision, pollination, pest control, and soil functions. However, lacking high spatial resolution images, their spatial heterogeneity remains poorly mapped, limiting our understanding of how these features support ecosystem services. This study presents a high-resolution dataset derived from unmanned aerial vehicle (UAV) RGB imagery for the semantic segmentation of diverse ground covers in urban community gardens. The dataset consists of 2,521 images processed into 24 orthomosaics, acquired in 2021–2022 at five garden locations in Munich, Germany. Each image (18.9–146.4 M px; 3.2–7.9 mm resolution) is manually annotated into eight ground-cover classes (grass, herb, litter, soil, stone, straw, wood, and woodchip). We evaluated deep-learning segmentation models, including UNet and DeepLabV3+. The DeepLabV3+ (overall accuracy = 93.2% and Intersection over Union = 69.4) achieved high classification accuracy in distinguishing these complex classes. This dataset is intended to support research on urban biodiversity, habitat modelling, garden management, remote sensing research, and can be integrated with other fine-scale datasets to advance sustainable urban green planning.

## Background & Summary

Urban gardens, though small in scale, comprise various ground covers such as soil, herbs, grass, different kinds of woods, and stones. These ground covers are not merely aesthetic but are components of the urban garden ecosystem that are related to biodiversity and ecosystem services^1–3^. The configuration and composition of these substrates directly influence pollinator populations, offering resources for pollinators like bees and butterflies^4,5^. Beyond pollination, various ground covers help pest control within urban gardens. The structure and heterogeneity of ground covers, including organic-rich areas from mulching or decomposed plant material, create favourable conditions for natural predators that help control pests, reducing the need for chemical pesticides^6,7^. These ground-cover substrates also contribute to soil health by enhancing nutrient cycling and preventing erosion. The enhanced organic matter content and diversity of soil fauna found in community gardens sustain soil structure and fertility while contributing to increased resilience against environmental disturbances^8–10^. Furthermore, varied ground covers substantially contribute to the overall urban ecological network. Urban gardens often serve as refugia and stepping stones for biodiversity in heavily built environments, connecting fragmented habitats and linking various ecological communities^11,12^. This connectivity enhances the potential for ecosystem services such as improved pollination and natural pest control, which are critical factors for the sustainable productivity of urban gardens^13–15^. Consequently, ground cover monitoring in these spaces is important for understanding and maintaining the ecological functions of urban gardens and their contributions to urban sustainability.

Advances in remote sensing technologies with distinct spatial, spectral, and geometric features have improved our ability to monitor environmental changes at local and regional scales. Nevertheless, monitoring ground covers in urban gardens presents several challenges. Urban gardens are highly heterogeneous in a relatively small spatial extent, characterized by numerous plant species and ground cover types. This heterogeneity, coupled with concerns about gardener privacy and the scarcity of open-access data, complicates ground cover monitoring tasks in urban gardens. Traditional field surveys and aerial photo interpretation, while valuable, are often laborious, time consuming and limited in their ability to accurately capture the fine-scale heterogeneity of ground covers^16,17^. Land use/land cover classification from remote sensing data is common at the landscape scale^18–22^. However, the spatial resolution of many remote sensing products is insufficient to capture the fine-scale complexity and variability in land use essential for assessing ecological functions in urban gardens^23,24^.

The small-scale spatial heterogeneity in urban gardens highlights the need for high-resolution remote sensing datasets that can capture the intricate variability of these spaces^23,25^. Unmanned Aerial Vehicles (UAVs) have emerged as a powerful tool in this context, due to their affordability, ease of use, ability to capture ultra-high-resolution images and rapid data collection, especially in inaccessible areas^26–31^. These images are rich in texture, shape, and spatial distribution information, enabling more accurate and detailed ground cover classification at a local scale^32^. One widely used approach for ground cover classification involves training models with labeled data, in which pixels are systematically categorized into different ground cover types. However, the complexity of mixed land uses in urban gardens requires careful sample selection and labeling to ensure accuracy. High inter- and intra-classes similarities and variations pose significant challenges, demanding substantial expertise and effort^33,34^. Despite these challenges, the use of high-resolution UAV technology for gathering comprehensive datasets is crucial for advancing our understanding and management of ground covers in heterogeneous urban gardens.

Traditional Machine Learning (ML) models, along with more advanced ML models based on deep neural networks have become increasingly important in land cover classification and feature detection^35–39^. Traditional ML models like Random Forest, maximum likelihood classifier, and Extreme Gradient Boosting are popular for their interpretability and robustness, particularly when dealing with smaller datasets^40^. A key factor for this is that these traditional ML classifiers typically achieve optimal performance after being trained on a large volume of data^41^. Traditional ML models encounter difficulties when handling raw data^42,43^, such as pixel values from images directly, requiring expert-designed feature extractors to transform raw data into meaningful representations^42^. Furthermore, these traditional classifiers often require manual feature extraction, which limits their effectiveness in complex environments like urban gardens^44,45^. Typically, traditional classifiers extract key visual features such as spectral, geometric, and textural characteristics using methods like scale-invariant feature transform, histogram of oriented gradient, and Gray-level co-occurrence matrix^40,46,47^. In contrast, deep learning based models excel in automatically learning features from data, making them especially well-suited for integrating diverse data sources and uncovering hidden patterns in land/ground cover classification.

In deep learning (DL) based classifiers, algorithms are structured to capture complex data patterns through multiple layers of processing using hierarchical architectures composed of nonlinear transformations^34,42^. These deep architectures, exemplified by fully convolutional and multitask networks, are designed to extract abstract representations from raw input data automatically, thereby effectively discerning fine-scale details in high spatial resolution imagery^17,48,49^. In general, DL based approaches are capable of automatically extracting features through multiple layers, gradually advancing from lower to higher levels of abstraction^42,44,50^. The effectiveness of DL based models in urban land/ground cover classification is strongly influenced by the quality of input data, particularly the band composition and spatial resolution of the images used. Particularly, high spatial resolution images enhance the ability of these models to learn relevant features related to land use, potentially improving classification accuracy even when fewer spectral bands are available^17,48^.

This study presents a high-resolution dataset and ML frameworks tailored to the unique challenges of urban garden ground cover classification. The specific objectives were to: (i) generate a manually annotated ground-truth dataset by labeling eight ground-cover classes including grass, herb, litter, soil, stone, straw, wood, and woodchip using RGB orthomosaic images captured by UAVs at a spatial resolution ranging from 3.2 to 7.9 mm per pixel; and ii) evaluate the classification accuracy of machine learning models using the annotated data. Together, the dataset and results provide a reference for future studies aiming to train, validate, or compare ground cover classification approaches in urban garden environments.

## Methods

### Image acquisition

**Fig. 1:**
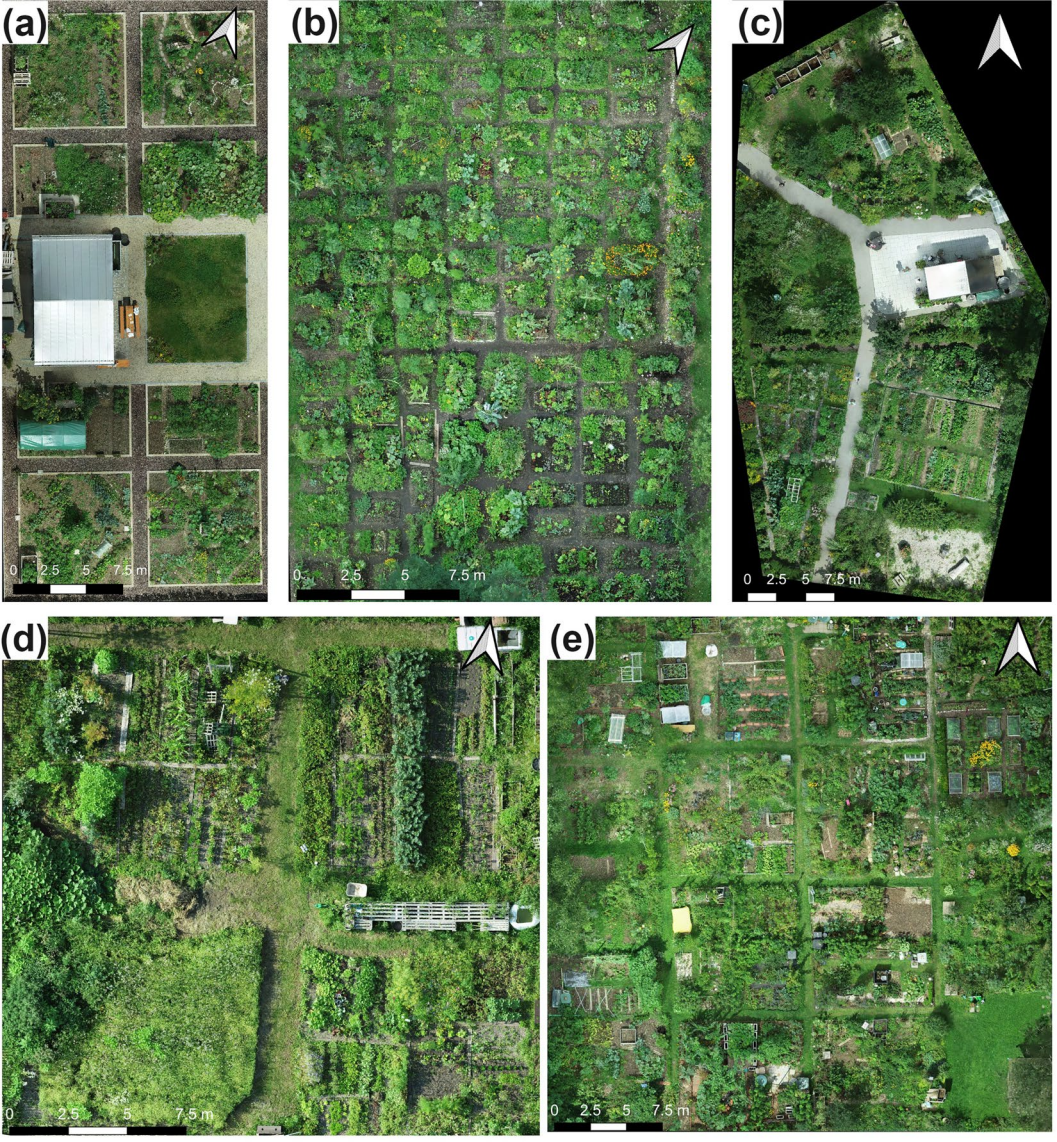
Example orthomosaics from five urban community gardens in Munich, Germany. (**a**) Freiluftgarten Freiham (FF), (**b**) Essbare Stadt (ESS), (**c**) StadtAcker (SA), (**d**) Karlsfeld Community Garden (KF), and (**e**) Sonnengarten Solln (SONN).

We collected a dataset of high-resolution RGB drone images from five urban community gardens in Munich, Germany: Essbare Stadt (ESS), Freiluftgarten Freiham (FF), Karlsfeld Community Garden (KF), StadtAcker (SA), and Sonnengarten Solln (SONN) (Fig. 1). These sites include a diverse range of ground cover types. Each garden was flown several times on different dates. In total, 24 UAV-orthomosaic images were produced, each capturing a full garden area.

Data collection occurred during 2021 and 2022. We conducted two sampling periods in 2021 (July and August) and three sampling periods in 2022 (May, July, and October). A site-level summary of orthomosaic acquisition dates is provided in Table 1. Full metadata for each orthomosaic (including exact acquisition time, spatial resolution, and flight information) are available in the dataset metadata (see Data Availability).

**Table 1:** Summary of orthomosaic image acquisitions by site.

Site name	n	2021 Jul	2021 Aug	2022 May	2022 Jul	2022 Oct
Essbare Stadt	5	2021-07-22	2021-08-17	2022-05-10	2022-07-19	2022-10-20
Freiluftgarten Freiham	5	2021-07-21	2021-08-17	2022-05-10	2022-07-19	2022-10-20
Karlsfeld	4	2021-07-21	2021-08-17	2022-05-10	—	2022-10-21
Sonnengarten Solln	5	2021-07-21	2021-08-17	2022-05-10	2022-07-19	2022-10-20
StadtAcker	5	2021-07-21	2021-08-17	2022-05-10	2022-07-19	2022-10-20

Columns represent the five sampling periods (two in 2021 and three in 2022). The image acquisition dates for each garden are reported as YYYY-MM-DD format, and n is the number of orthomosaics available for that site.

Data acquisition was carried out using a DJI Phantom 4 RTK UAV equipped with a high-resolution RGB camera (DJI, Shenzhen, China) and an RTK (Real-Time Kinematic) corrections system using SAPOS (Bavaria), enabling centimeter-level georeferencing without the use of ground control points (GCPs). A total of 2,521 raw RGB images were collected across five garden sites and mosaicked into 24 orthomosaics. Flights were planned with a 90% front overlap and 90% side overlap to ensure full coverage and reliable image matching. To minimize shadow effects from low sun angles, all flights were conducted in clear-sky conditions between 12:00 and 15:00 local time, when solar elevation is highest. Sky conditions were not systematically quantified and are therefore not reported per flight. Even with midday flights, some local shadows are unavoidable due to trees and nearby buildings.

**Fig. 2:**
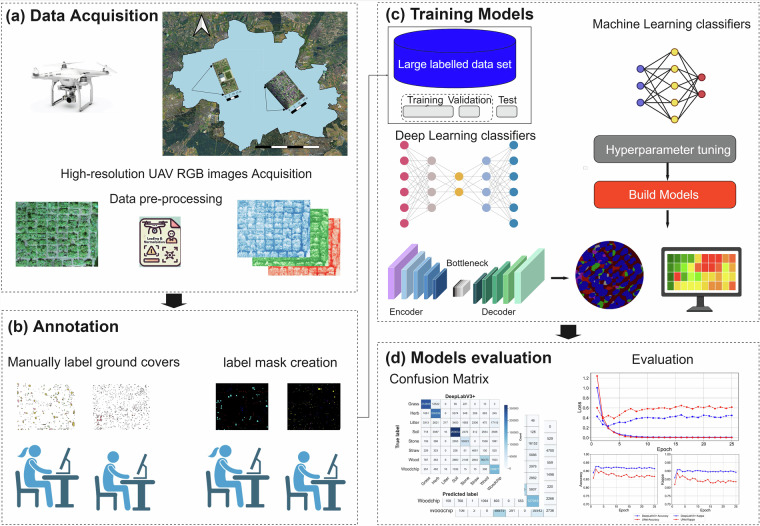
Workflow for ground-cover segmentation using high-resolution UAV RGB imagery. (**a**) RGB images are collected with a UAV and processed into orthomosaics. (**b**) Ground cover types are manually labeled to create reference labels and class masks. (**c**) Machine learning segmentation models are trained using the orthomosaics and labeled masks. (**d**) Model performance is evaluated by examining the confusion matrix and accuracy metrics to assess classification reliability.

Following data collection, the raw images underwent a preprocessing workflow using Agisoft Metashape (©2023 Agisoft LLC). This process involved image alignment and calibration. Radiometric calibration was performed to correct for variations in illumination and standardize image brightness across the dataset. The RTK-geotagged image positions were used in Agisoft Metashape for image alignment and georeferencing (one per raw UAV image; n = 2,521), and no separate ground control points were used. Finally, the aligned and calibrated images were stitched together to generate high-resolution orthomosaics Fig. 2.

### Manual image annotation

We created label masks using ENVI 6.0 (NV5 Geospatial, Broomfield, CO, USA) software. Regions of interest (ROIs) were manually delineated to represent eight distinct ground cover types: grass, herb, litter, soil, stone, straw, wood, and woodchip. We selected these eight classes because they represent the main ground-cover types observed across the gardens. Some classes are related in a botanical or material group (e.g., grass is herbaceous vegetation, and woodchip is processed wood), but they represent different ecological functions, garden management practices, and visual patterns in the imagery, requiring distinct considerations to capture their unique contributions to ecosystem services (e.g., pollination, pest control, soil health, and habitat provision). We manually drew polygons based on visible cues such as color, texture, and context in the orthomosaics. Multiple polygons were defined for each class on RGB orthomosaic images to capture spatial variability and ensure representative sampling (Table 2). Annotation was carried out by team members with experience from on-site surveys in Munich urban community gardens, which helped refine the class definitions and interpretation rules and improve consistency during labelling^51^. The polygons within each ROI were then saved and labeled according to their corresponding ground cover class.

**Table 2:** Summary of annotation statistics for each dataset split.

Metric	Train	Validation	Test
**Total Number of Labeled Pixels**	3063488	1452517	1072618
**Total Number of Polygons**	26386	10564	10586
**Average Number of Polygons per orthomosaic**	1884.7	2112.8	2117.2
**Total Number of Pixels Per Class**			
**Grass**	571449	309453	246573
**Herb**	975525	341117	216029
**Litter**	35845	25832	31460
**Soil**	745669	422262	277257
**Stone**	126584	75056	62860
**Straw**	42741	10645	6142
**Wood**	240461	144103	101001
**Woodchip**	325214	124049	131296
**Total Number of Polygons Per Class**			
**Grass**	5053	1392	1346
**Herb**	9425	2770	3457
**Litter**	868	238	1054
**Soil**	4028	2393	1517
**Stone**	2859	1506	1559
**Straw**	874	505	148
**Wood**	2064	985	1040
**Woodchip**	1215	775	465

The table reports the total number of labeled pixels and their distribution across the eight ground-cover classes and the total number of polygons overall and per class.

**Fig. 3:**
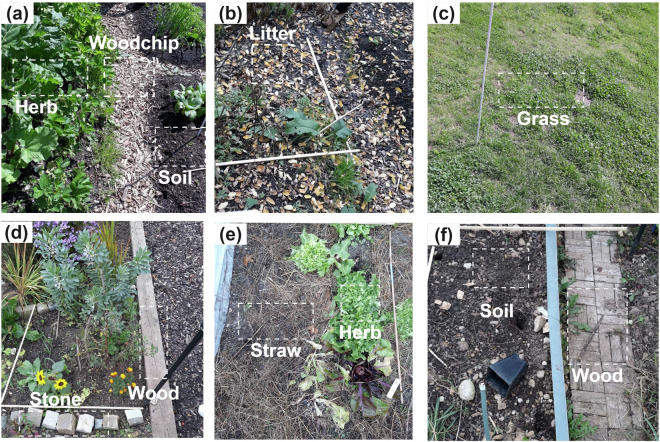
Smartphone field photos showing examples of the eight ground-cover classes. Dashed boxes indicate representative regions corresponding to the annotation classes (grass, herb, litter, soil, stone, straw, wood, and woodchip) within mixed garden scenes.

The masks should be treated as reference labels from image interpretation, not as pixel-by-pixel field-verified ground truth. Some confusion is still possible for classes with similar appearance (e.g., litter vs. woodchip in shadowed areas). To help users understand the class definitions, Fig. 3 shows urban gardens field scenes with example regions for each class. We also provide site-level size statistics and class distributions in Table 3.

**Table 3:** Site-level spatial statistics and class distributions.

Garden	Freiluftgarten Freiham	Karlsfeld	Sonnengarten Solln	Stadtacker	Essbare Stadt
Area (m^2^)	778.1	519.5	1280.6	1065.5	575.1
Width (m)	18.7	22.5	35.5	—	20.5
Height (m)	41.5	23.1	36.1	—	28.2
Grass (%)	5.32	33.95	27.38	26.58	15.83
Herb (%)	11.35	21.61	28.53	33.88	45.23
Litter (%)	0.00	0.59	2.22	7.93	0.21
Soil (%)	31.83	28.89	24.60	14.62	24.57
Stone (%)	9.41	0.58	3.86	8.85	0.87
Straw (%)	0.11	1.99	1.82	2.12	0.05
Wood (%)	16.28	12.39	5.83	5.90	2.44
Woodchip (%)	25.69	0.00	5.76	0.13	10.81

For each garden, we report the average orthomosaic size; width and height in meter (m), and area in square meters (m^2^) using only the valid image part and averaged over all flights for that site. The class percentages come from the label masks and show the fraction of labeled pixels in each class.

### Dataset splits for annotation

The annotated dataset consists of 24 RGB orthomosaics (in GeoTIFF format) and 24 corresponding label masks. These data were divided into three subsets: training, validation, and testing. We used an orthomosaic-level split, meaning each orthomosaic (one flight over one garden on one date) and its corresponding label mask, was assigned to only one subset (train/validation/test), so there is no overlap across subsets. Each subset (training, validation, and test) includes examples of all eight ground cover classes, and all five gardens are represented in each subset. Therefore, the test set evaluates generalization to new orthomosaics and new dates, rather than to completely unknown garden sites. This split reduces spatial leakage between training, validation, and testing that could otherwise inflate accuracy^52^. Full split details are listed in the dataset metadata file (see Data Availability).

## Data Records

The complete UAV imagery dataset is publicly available on Zenodo^53^. The dataset is organized into training, validation, and test subsets, each in its own folder including: Train/14 RGB orthomosaic image files (GeoTIFF) and their 14 corresponding labeled mask files (GeoTIFF). Val/5 RGB image files +5 labeled mask files. Test/5 RGB image files +5 labeled mask files. Each RGB image is a high-resolution, three-band orthomosaic with a spatial resolution below 1 cm (ranging from 3.2 to 7.9 mm per pixel) and pixel counts ranging from 18.9 to 146.4 million (EPSG:25832). The corresponding reference labels are a single-band GeoTIFF of identical dimensions, where each pixel’s value represents one of eight class labels (grass, herb, soil and etc.). These annotations serve as reference labels for semantic segmentation tasks. File names follow a clear convention, including capture date and site, for example, RGB_2021-07-21 garden Freiluftgarten Freiham.tif for an orthomosaic collected on July 21, 2021, at the Freiluftgarten Freiham garden, with its labeled mask named RGB_2021-07-21 garden Freiluftgarten Freiham_Labeled.tif.

Moreover, to support deep learning applications, a patch-based version of the dataset is included (explained in section ‘Machine Learning Models based on deep learning’). The patch data are organized in a “patches” directory, with two subfolders: “images” and “masks.” The “images” folder contains the cropped patches derived from the UAV orthomosaic imagery, and the “masks” folder holds the corresponding cropped labeled GeoTIFF files. These image patches are organized in the same folder structure (Train/, Val/, Test/) and follow a naming convention that appends a patch index to the original file name, e.g., RGB_2021-07-21 garden Freiluftgarten Freiham_patch_0.tif. Corresponding label patches are similarly named, ensuring traceability.

In addition to the primary image and mask files, the repository includes a comprehensive metadata file (dataset_metadata.csv). This CSV file contains records for each image and its label mask, with key attributes such as filename, image dimensions, pixel count, spatial resolution, number of bands, and coordinate reference system. Importantly, it also documents supplementary details derived from processing and flight logs. For each image, metadata fields capture Agisoft processing information (e.g., alignment, dense cloud settings, DEM generation) and flight properties (e.g., flight height, GPS type, image overlap, acquisition time).

## Technical Validation

### Classification based on machine learning

The traditional learning based machine learning models used in this study include a Random Forest (RF) classifier, an eXtreme Gradient Boosting (XGBoost) classifier, and a Quadratic Discriminant Analysis (QDA) model used as a Maximum Likelihood Classifier (MLC). The implementations were done in Python 3.12, using scikit-learn (1.3.2). Code and dependencies are available in the GitHub repository (see Code Availability). Full model settings and tuning details are provided in the Supplementary Information.

Each RGB orthomosaic has three channels, and corresponding label masks contain nine distinct ground cover classes. However, one of these is a background class labeled as 0 and excluded from analysis, resulting in eight classes. The corresponding label images are located based on predetermined filename patterns.

A patch-based approach was used to capture spatial context. A single pixel does not contain information about its surroundings, making it difficult for the model to distinguish between classes, especially for a large number of pixels in complex scenes with spectrally similar characteristics^54,55^. By using small patches around each pixel, the classifier gains important information, such as texture, patterns, local details, and spatial relationships, which helps in distinguishing between different classes more reliably. For each labeled pixel (non-background), we extract a 11 × 11 window from the RGB orthomosaic centered on that pixel (stride = 1 pixel). In addition, a reflective padding is used to handle edge regions, ensuring that the patches have a uniform size. Each patch, which captures local spatial and spectral information (11 × 11 × 3), is then flattened into a one-dimensional feature vector and the class label is taken from the center pixel in the label mask.

### Classification based on deep learning

For deep learning based segmentation, maintaining the spatial structure of the input images is critical. In contrast to machine learning approaches based on traditional learning, which extract and flatten small 11 × 11 pixels around central pixel to compensate for their limited feature extraction capabilities, DL models utilize convolutional neural networks (CNNs) to automatically learn hierarchical features directly from two-dimensional inputs^56^. Due to the large dimensions of the orthomosaic (which can span millions of pixels in both height and width) processing entire images is impractical because of memory limitations. Therefore, a large set of smaller image patches was extracted for model training. We implemented a sliding window cropping script that reads orthomosaics and their corresponding label masks using the rasterio library. Each image was cropped into overlapping patches of 512 × 512 pixels with a stride of 256 pixels. This patch-based approach effectively captures both fine details and broader contextual information while facilitating the reconstruction of full-image predictions. It also allows the CNN to predict a class label for every pixel in each patch by learning patterns (e.g., edges, textures, object boundaries), thereby enhancing segmentation accuracy in complex scenes like urban gardens with spectrally similar classes^42^.

In this work, we used two established DL frameworks, UNet^57,58^ and DeepLabV3+^59^, each incorporating a ResNet50 encoder, and pre-trained on the ImageNet^60,61^ dataset to enhance feature extraction and leverage transfer learning. Both models were trained using a supervised learning approach on the prepared training set of image patches. We trained for 25 epochs, using a mini-batch size of 16 patches per iteration. The Adam optimizer^62^ was used to update model weights, with dynamic learning rate starting at 1 × 10^−5^, using a patience of 5 epochs and a reduction factor of 0.1. This low learning rate was chosen to cautiously fine-tune the pre-trained ResNet50 encoder weights without causing drastic changes early in training. The model’s weights after each epoch were saved if the validation loss had improved (decreased) compared to previous epochs. The final model selected for testing was the one with the lowest validation loss observed during the 25 epochs^63^. Full architecture descriptions, training settings, and additional benchmarking outputs (Figure S2, S3) are provided in the Supplementary Information.

### Testing annotation through pixel-wise classification

After training, the best models were applied to generate ground cover maps for each garden environment within the test subset. We evaluated the model’s performance using a suite of standard accuracy metrics, computed by comparing the predicted ground covers against the label masks of the test set on a per-pixel basis.

**Fig. 4:**
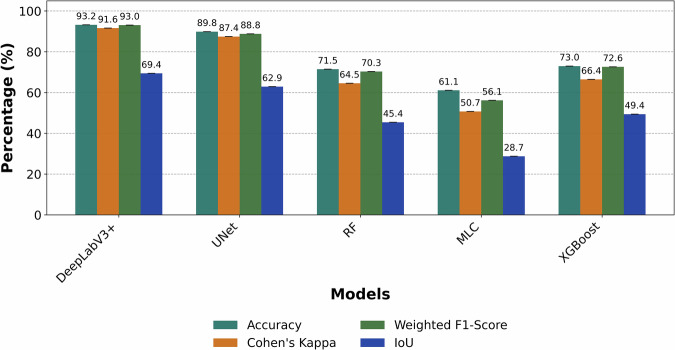
Model performance comparison across classifiers. Overall Accuracy, Cohen’s Kappa, weighted F1-score, and Intersection-over-Union (IoU) are shown for DeepLabV3 + , UNet, Random Forest (RF), Maximum Likelihood Classifier (MLC), and Extreme Gradient Boosting (XGBoost).

To assess the segmentation performance, a set of comprehensive metrics was employed, which included overall pixel accuracy, Cohen’s Kappa, and Intersection over Union (IoU). We also calculated weighted F1-score^64,65^, which is a performance metric that computes the harmonic mean of precision and recall for each of eight foreground classes, subsequently averaged with weights proportional to the pixel count per class (Fig. 4). As shown in Fig. 4, the deep-learning models achieved higher overall scores than the traditional baselines. DeepLabV3 + obtained the highest values (Accuracy = 93.2%, Kappa = 91.6%, weighted F1 = 93.0%, IoU = 69.4%), followed by UNet (89.8%, 87.4%, 88.8%, 62.9%). Among the traditional models, XGBoost performed best (73.0%, 66.4%, 72.6%, 49.4%).

**Fig. 5:**
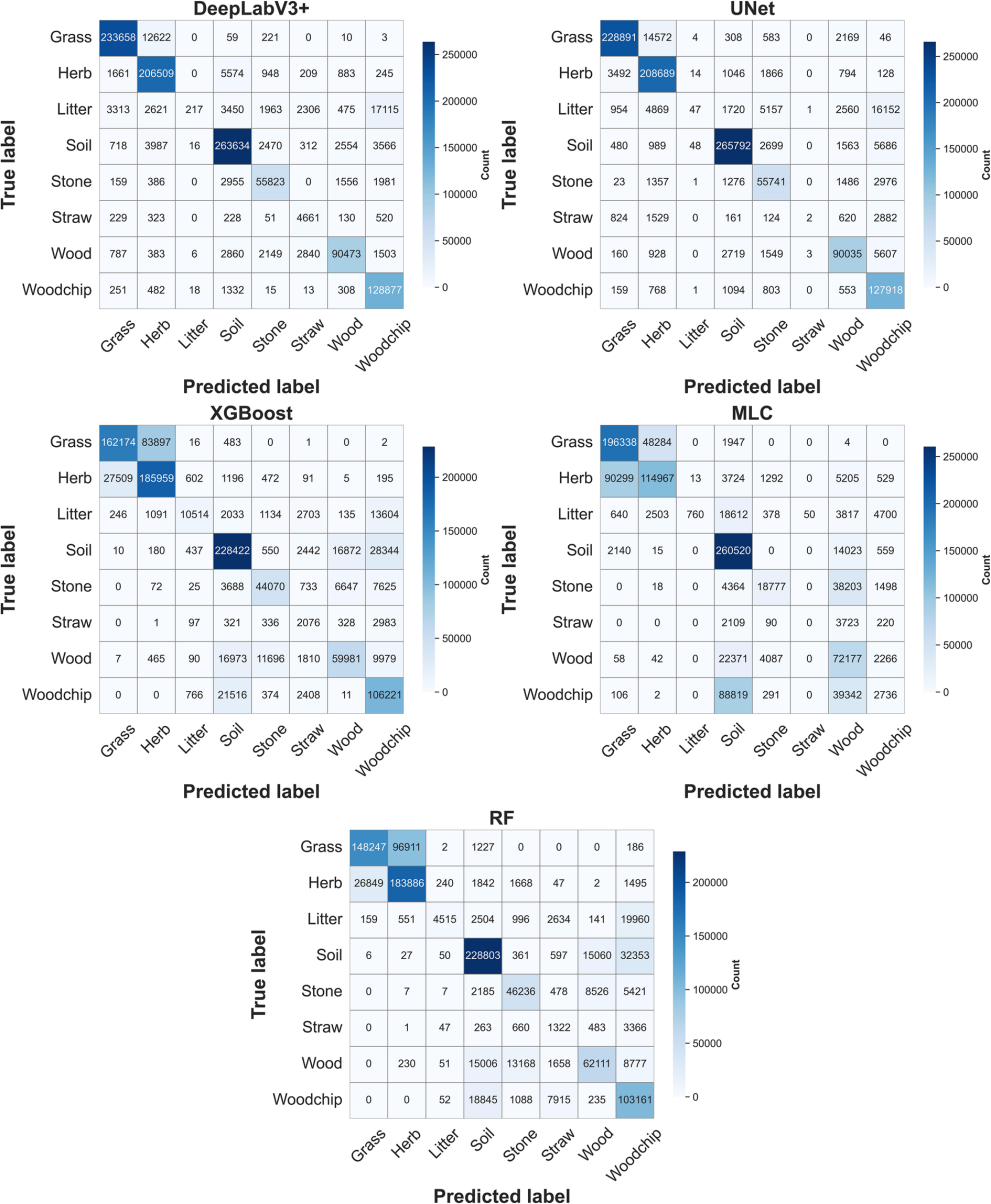
Confusion matrices for five models on the test set. Confusion matrices are shown for DeepLabV3+, UNet, Extreme Gradient Boosting (XGBoost), Maximum Likelihood Classifier (MLC), and Random Forest (RF) across the eight ground-cover classes. Cell values and color intensity indicate the number of pixels assigned to each true–predicted class combination.

To provide further insight into the model’s performance, a confusion matrix was generated (Fig. 5) for the test set, summarizing the distribution of predicted classes versus true classes. By examining this matrix, we can see the types of misclassification errors, for example, whether certain ground cover classes are frequently confused with each other or mislabeled. This matrix was used to derive class-wise Producer’s Accuracy (PA) and User’s Accuracy (UA)^37^ (Table 4), as well as class-wise IoU values (Figure S1). The mean IoU was calculated as the average of the class-wise IoU scores across the eight classes. These metrics collectively reveal patterns of misclassification and highlight frequent inter-class confusions (e.g., between spectrally or texturally similar ground covers). Class-wise results (Table 4) show that performance varies by ground-cover class. In general, classes such as grass, herb, and soil were classified more reliably, while litter and straw had lower UA and PA in several models. In addition, the confusion matrices show that many errors occur between visually similar classes (for example, grass vs. herb and soil vs. stone), with fewer such confusions in the deep-learning models than in the traditional baselines.

**Table 4:** Per-class User’s accuracy (UA; %) and producer’s accuracy (PA; %) for ground-cover classification across five models.

Models	DeepLabV3+		UNet		XGBoost		RF		MLC	
Classes	UA	PA	UA	PA	UA	PA	UA	PA	UA	PA
Grass	97.79	95.84	97.05	91.66	83.51	54.89	83.57	45.38	66.73	70.59
Herb	93.55	93.93	87.24	95.85	61.26	86.00	58.01	88.20	62.78	57.28
Litter	75.64	47.33	30.99	0.16	81.12	29.92	87.18	9.26	94.12	1.59
Soil	93.70	95.81	96.85	94.64	83.78	85.42	83.91	87.95	68.64	93.32
Stone	86.41	90.10	80.21	85.69	71.35	71.09	71.97	70.94	64.38	32.84
Straw	53.68	67.10	35.06	0.12	19.34	40.70	10.73	18.22	0.00	0.00
Wood	95.07	88.76	89.34	87.84	73.03	61.08	76.69	60.30	36.24	68.92
Woodchip	89.58	97.33	78.10	95.80	67.92	77.45	65.97	79.02	17.97	1.09

User’s accuracy and producer’s accuracy are reported for each ground-cover class (Grass, Herb, Litter, Soil, Stone, Straw, Wood, and Woodchip) on the independent test dataset for DeepLabV3 + , U-Net, XGBoost, Random Forest (RF), and Maximum Likelihood Classifier (MLC).

**Fig. 6:**
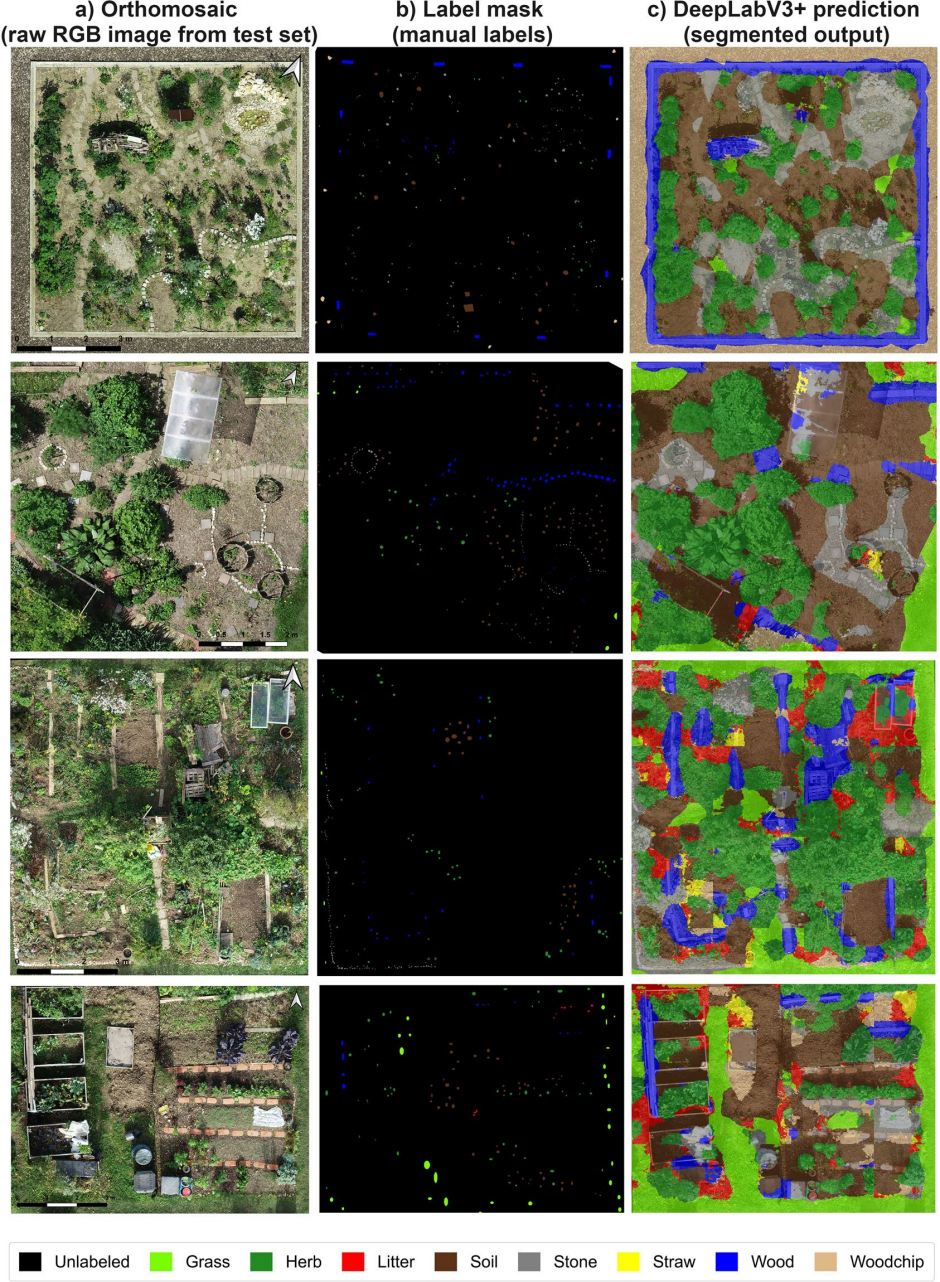
Visual comparison of ground cover semantic segmentation results on selected cropped regions from test-set orthomosaics. Each triplet shows: (**a**) a cropped orthomosaic RGB tile from the test dataset, (**b**) the corresponding label mask with eight ground-cover classes (grass, herb, litter, soil, stone, straw, wood, woodchip), and (**c**) the predicted segmentation map generated by the DeepLabV3 + model.

As an illustrative usability example of the released dataset and code, Fig. 6 shows examples from the test set, including the RGB orthomosaic, the label mask, and the corresponding DeepLabV3 + prediction. For clearer visualization, only small cropped regions of the test orthomosaics are displayed.

## Usage Notes

In the context of semantic segmentation for high-resolution aerial images of heterogeneous urban gardens, this study shows a proof of concept that drone-acquired centimeter-spatial resolution images in conjunction with advanced deep learning models enabled mapping of eight distinct urban garden ground cover types critical for urban biodiversity monitoring. While the models evaluated, particularly DeepLabV3+ and the UNet exhibit promising performance, a few challenges remain. These limitations can significantly highlight the importance of this dataset and the practical application of these models in biodiversity surveys and ecological assessments.

Data requirements: The acquisition and annotation of labeled datasets for ecological image segmentation can be resource-intensive and time-consuming. For wildlife classification, 150–1,000 camera trap images per class are recommended for classification^66^. For segmentation, we estimate a similar range of 150–1,000 annotated polygons per class, reflecting typical UAV-based segmentation needs. To assess whether the annotation quality is consistent, we additionally tested a reduced subset of 10 orthomosaic images (6 training, 2 validation, and 2 testing), containing 2,613,994 labeled pixels derived from 5,879 labeled polygons across 8 ground-cover classes. This subset experiment showed results that were consistent with the full dataset experiments, suggesting that the quality of the annotation is consistent. However, expanding the dataset would likely enhance model robustness and generalization, particularly for capturing complex ecological features.

Annotation limitation: The label masks were produced by manual interpretation of UAV RGB orthomosaics. Annotators visited the same gardens and conducted vegetation survey work, which supported class definitions and consistent decisions. However, the label masks are still not validated pixel-by-pixel through on-site verification. Users should expect some label uncertainty where classes look similar or are partly hidden due to image resolution limits (e.g., shadowed areas or mixed materials), and should consider this when training and evaluating models.

Computational resources: Training deep learning models for ecological image segmentation, such as our orthomosaic-based urban garden monitoring, requires substantial computational resources, including high-performance GPUs and significant memory capacity^56^. This requirement can pose a barrier for researchers and practitioners with limited access to advanced computational infrastructure. Reducing computational demands could involve developing lightweight architectures or distributed processing pipelines, making deep learning approaches more accessible to practitioners with limited hardware resources. For example, distributed frameworks like Horovod^67^ could reduce computational demands while maintaining segmentation accuracy.

Limited spectral range: Reliance on UAV-derived high-resolution RGB imagery without the near-infrared or red-edge bands available in multispectral and hyperspectral sensors restricts spectral information and therefore hinders the ability of the models to discriminate different ground cover and certain vegetation types.

Class imbalance: Due to class imbalance in the training dataset, the segmentation models often focus on the most abundant ground cover types, resulting in suboptimal accuracy for less frequent yet ecologically pivotal classes (e.g., litter, straw). This misrepresentation can hinder broader ecological insights, as these uncommon ground cover types frequently play critical roles in habitat provision and nutrient cycling within urban gardens. However, similar results were found when testing on a subset, suggesting that the class imbalance does not affect the model results significantly.

Heterogeneity and scale: Due to the diverse mix of plants, soil, and anthropogenic materials within urban gardens, each ground-cover class can appear spectrally different across locations and even within the same site. This variability poses a challenge for semantic segmentation models, to maintain accurate segmentation at fine spatial scales^68^.

## Supplementary information

Supplementary information

## Data Availability

The dataset generated by the current study is available on Zenodo^53^ (10.5281/zenodo.18757882).

## Code availability

The code is available on GitHub (https://github.com/paglab/ugc-mapping).

## References

[1] Jha, S. *et al*. Multiple ecosystem service synergies and landscape mediation of biodiversity within urban agroecosystems. *Ecol. Lett.* **26**, 369–383, 10.1111/ELE.14146 (2023). 36691722 10.1111/ele.14146

[2] Lin, B. B. *et al*. COVID‐19 gardening could herald a greener, healthier future. *Front. Ecol. Environ.* **19**, 491–493, 10.1002/FEE.2416 (2021). 34899093 10.1002/fee.2416 PMC8652897

[3] Cabral, I. *et al*. Ecosystem services of allotment and community gardens: A Leipzig, Germany case study. *Urban For. Urban Green.* **23**, 44–53, 10.1016/J.UFUG.2017.02.008 (2017).

[4] Klein, A. M. *et al*. Importance of pollinators in changing landscapes for world crops. *Proc. R. Soc. B Biol. Sci.* **274**, 303–313, 10.1098/rspb.2006.3721 (2007). 10.1098/rspb.2006.3721 PMC1702377 17164193

[5] Plascencia, M. & Philpott, S. M. Floral abundance, richness, and spatial distribution drive urban garden bee communities. *Bull. Entomol. Res.* **107**, 658–667, 10.1017/S0007485317000153 (2017). 28245886 10.1017/S0007485317000153

[6] Reganold, J. P., Glover, J. D., Andrews, P. K. & Hinman, H. R. Sustainability of three apple production systems. *Nature* **410**, 926–930, 10.1038/35073574 (2001). 11309616 10.1038/35073574

[7] Tresch, S. *et al*. Direct and indirect effects of urban gardening on aboveground and belowground diversity influencing soil multifunctionality. *Sci. Rep.* **9**, 9769, 10.1038/S41598-019-46024-Y (2019). 31278335 10.1038/s41598-019-46024-y PMC6611818

[8] Igalavithana, A. D. *et al*. Assessment of soil health in urban agriculture: Soil enzymes and microbial properties. *Sustainability* **9**, 310, 10.3390/su9020310 (2017).

[9] Salomon, M. J., Watts-Williams, S. J., McLaughlin, M. J. & Cavagnaro, T. R. Urban soil health: A city-wide survey of chemical and biological properties of urban agriculture soils. *J. Clean. Prod.* **275**, 122900, 10.1016/J.JCLEPRO.2020.122900 (2020). 32834569 10.1016/j.jclepro.2020.122900 PMC7362792

[10] Wander, M. M. *et al*. Developments in Agricultural Soil Quality and Health: Reflections by the Research Committee on Soil Organic Matter Management. *Front. Environ. Sci.* **7**, 1–9, 10.3389/fenvs.2019.00109 (2019).

[11] App, M., Strohbach, M. W., Schneider, A. K. & Schröder, B. Making the case for gardens: Estimating the contribution of urban gardens to habitat provision and connectivity based on hedgehogs (Erinaceus europaeus). *Landsc. Urban Plan.* **220**, 104347, 10.1016/J.LANDURBPLAN.2021.104347 (2022).

[12] Graffigna, S., González-Vaquero, R. A., Torretta, J. P. & Marrero, H. J. Importance of urban green areas’ connectivity for the conservation of pollinators. *Urban Ecosyst.* **27**, 417–426, 10.1007/S11252-023-01457-2 (2024).

[13] Taylor, J. R. & Lovell, S. T. Urban home gardens in the Global North: A mixed methods study of ethnic and migrant home gardens in Chicago, IL. *Renew. Agric. Food Syst.* **30**, 22–32, 10.1017/S1742170514000180 (2015).

[14] Davies, Z. G. *et al*. A national scale inventory of resource provision for biodiversity within domestic gardens. *Biol. Conserv.* **142**, 761–771, 10.1016/j.biocon.2008.12.016 (2009).

[15] Felderhoff, J., Gathof, A. K., Buchholz, S. & Egerer, M. Vegetation complexity and nesting resource availability predict bee diversity and functional traits in community gardens. *Ecol. Appl.* **33**, e2759, 10.1002/eap.2759 (2023). 36217895 10.1002/eap.2759

[16] Egerer, M. H., Wagner, B., Lin, B. B., Kendal, D. & Zhu, K. New methods of spatial analysis in urban gardens inform future vegetation surveying. *Landsc. Ecol.* **35**, 761–778, 10.1007/S10980-020-00974-1 (2020).

[17] Huang, B., Zhao, B. & Song, Y. Urban land-use mapping using a deep convolutional neural network with high spatial resolution multispectral remote sensing imagery. *Remote Sens. Environ.* **214**, 73–86, 10.1016/J.RSE.2018.04.050 (2018).

[18] Giang, T. L. *et al*. Coastal landscape classification using convolutional neural network and remote sensing data in Vietnam. *J. Environ. Manage.* **335**, 117537, 10.1016/J.JENVMAN.2023.117537 (2023). 36842358 10.1016/j.jenvman.2023.117537

[19] Deshpande, P., Belwalkar, A., Dikshit, O. & Tripathi, S. Historical land cover classification from CORONA imagery using convolutional neural networks and geometric moments. *Int. J. Remote Sens.* **42**, 5148–5175, 10.1080/01431161.2021.1910365 (2021).

[20] Hermosilla, T., Wulder, M. A., White, J. C. & Coops, N. C. Land cover classification in an era of big and open data: Optimizing localized implementation and training data selection to improve mapping outcomes. *Remote Sens. Environ.* **268**, 112780, 10.1016/J.RSE.2021.112780 (2022).

[21] Bansal, K. & Tripathi, A. K. Dual level attention based lightweight vision transformer for streambed land use change classification using remote sensing. *Comput. Geosci.* **191**, 105676, 10.1016/J.CAGEO.2024.105676 (2024).

[22] Yao, J., Zhang, B., Li, C., Hong, D. & Chanussot, J. Extended Vision Transformer (ExViT) for Land Use and Land Cover Classification: A Multimodal Deep Learning Framework. *IEEE Trans. Geosci. Remote Sens*. **61**, 10.1109/TGRS.2023.3284671 (2023).

[23] Wagner, B. & Egerer, M. Application of UAV remote sensing and machine learning to model and map land use in urban gardens. *J. Urban Ecol.* **8**, 1–12, 10.1093/jue/juac008 (2022).

[24] Seitz, B. *et al*. Land sharing between cultivated and wild plants: urban gardens as hotspots for plant diversity in cities. *Urban Ecosyst.* **25**, 927–939, 10.1007/S11252-021-01198-0 (2022).

[25] Goddard, M. A., Dougill, A. J. & Benton, T. G. Scaling up from gardens: biodiversity conservation in urban environments. *Trends Ecol. Evol.* **25**, 90–98, 10.1016/j.tree.2009.07.016 (2010). 19758724 10.1016/j.tree.2009.07.016

[26] Anderson, K. & Gaston, K. J. Lightweight unmanned aerial vehicles will revolutionize spatial ecology. *Front. Ecol. Environ.* **11**, 138–146, 10.1890/120150 (2013).

[27] Pádua, L. *et al*. UAS, sensors, and data processing in agroforestry: A review towards practical applications. *Int. J. Remote Sens.* **38**, 2349–2391, 10.1080/01431161.2017.1297548 (2017).

[28] Colomina, I. & Molina, P. Unmanned aerial systems for photogrammetry and remote sensing: A review. *ISPRS J. Photogramm. Remote Sens.* **92**, 79–97, 10.1016/J.ISPRSJPRS.2014.02.013 (2014).

[29] Toth, C. & Jóźków, G. Remote sensing platforms and sensors: A survey. *ISPRS J. Photogramm. Remote Sens.* **115**, 22–36, 10.1016/J.ISPRSJPRS.2015.10.004 (2016).

[30] Pajares, G. Overview and current status of remote sensing applications based on unmanned aerial vehicles (UAVs). *Photogramm. Eng. Remote Sens.* **81**, 281–330, 10.14358/PERS.81.4.281 (2015).

[31] Aasen, H., Honkavaara, E., Lucieer, A. & Zarco-Tejada, P. J. Quantitative remote sensing at ultra-high resolution with UAV spectroscopy: A review of sensor technology, measurement procedures, and data correction workflows. *Remote Sens.* **10**, 1091, 10.3390/rs10071091 (2018).

[32] Cui, H. *et al*. Knowledge evolution learning: A cost-free weakly supervised semantic segmentation framework for high-resolution land cover classification. *ISPRS J. Photogramm. Remote Sens.* **207**, 74–91, 10.1016/J.ISPRSJPRS.2023.11.015 (2024).

[33] Zhu, Q. *et al*. Knowledge-guided land pattern depiction for urban land use mapping: A case study of Chinese cities. *Remote Sens. Environ.* **272**, 112916, 10.1016/j.rse.2022.112916 (2022).

[34] Du, S., Du, S., Liu, B. & Zhang, X. Mapping large-scale and fine-grained urban functional zones from VHR images using a multi-scale semantic segmentation network and object based approach. *Remote Sens. Environ.* **261**, 112480, 10.1016/J.RSE.2021.112480 (2021).

[35] Li, Z., Weng, Q., Zhou, Y., Dou, P. & Ding, X. Learning spectral-indices-fused deep models for time-series land use and land cover mapping in cloud-prone areas: The case of Pearl River Delta. *Remote Sens. Environ.* **308**, 114190, 10.1016/J.RSE.2024.114190 (2024).

[36] Ghosh, A., Sharma, R. & Joshi, P. K. Random forest classification of urban landscape using Landsat archive and ancillary data: Combining seasonal maps with decision level fusion. *Appl. Geogr.* **48**, 31–41, 10.1016/J.APGEOG.2014.01.003 (2014).

[37] Chowdhury, M. S. Comparison of accuracy and reliability of random forest, support vector machine, artificial neural network and maximum likelihood method in land use/cover classification of urban setting. *Environ. Challenges* **14**, 100800, 10.1016/J.ENVC.2023.100800 (2024).

[38] Christovam, L. E., Pessoa, G. G., Shimabukuro, M. H. & Galo, M. L. B. T. Land use and land cover classification using hyperspectral imagery: Evaluating the performance of spectral angle mapper, support vector machine and random forest. *Int. Arch. Photogramm. Remote Sens. Spatial Inf. Sci.* **XLII-2/W13**, 1841–1847, 10.5194/isprs-archives-XLII-2-W13-1841-2019 (2019).

[39] Kavzoglu, T. & Bilucan, F. Effects of auxiliary and ancillary data on LULC classification in a heterogeneous environment using optimized random forest algorithm. *Earth Sci. Informatics* **16**, 415–435, 10.1007/S12145-022-00874-9 (2023).

[40] Li, Z. *et al*. Deep learning for urban land use category classification: A review and experimental assessment. *Remote Sens. Environ.* **311**, 114290, 10.1016/J.RSE.2024.114290 (2024).

[41] Alom, M. Z. *et al*. A state-of-the-art survey on deep learning theory and architectures. *Electron.* **8**, 292, 10.3390/electronics8030292 (2019).

[42] LeCun, Y., Bengio, Y. & Hinton, G. Deep learning. *Nature* **521**, 436–444, 10.1038/nature14539 (2015). 26017442 10.1038/nature14539

[43] Bengio, Y., Courville, A. & Vincent, P. Representation learning: A review and new perspectives. *IEEE Trans. Pattern Anal. Mach. Intell.* **35**, 1798–1828, 10.1109/TPAMI.2013.50 (2013). 23787338 10.1109/TPAMI.2013.50

[44] Ball, J. E., Anderson, D. T. & Chan, C. S. Comprehensive survey of deep learning in remote sensing: theories, tools, and challenges for the community. *J. Appl. Remote Sens.* **11**, 042609, 10.1117/1.JRS.11.042609 (2017).

[45] Yuan, Q. *et al*. Deep learning in environmental remote sensing: Achievements and challenges. *Remote Sens. Environ.* **241**, 111716, 10.1016/j.rse.2020.111716 (2020).

[46] Dalal, N. & Triggs, B. Histograms of oriented gradients for human detection. In *Proc. IEEE Conf. Comput. Vis. Pattern Recognit*. 886–893, 10.1109/CVPR.2005.177 (2005).

[47] Guo, Z., Wen, J. & Xu, R. A Shape and Size Free-CNN for Urban Functional Zone Mapping With High-Resolution Satellite Images and POI Data. *IEEE Trans. Geosci. Remote Sens*. **61**, 10.1109/TGRS.2023.3320658 (2023).

[48] Fan, Y., Ding, X., Wu, J., Ge, J. & Li, Y. High spatial-resolution classification of urban surfaces using a deep learning method. *Build. Environ.* **200**, 107949, 10.1016/J.BUILDENV.2021.107949 (2021).

[49] Bergado, J. R., Persello, C. & Stein, A. LAND USE CLASSIFICATION USING DEEP MULTITASK NETWORKS. *Int. Arch. Photogramm. Remote Sens. Spat. Inf. Sci.* **XLIII-B3-2020**, 17–21, 10.5194/ISPRS-ARCHIVES-XLIII-B3-2020-17-2020 (2020).

[50] Reichstein, M. *et al*. Deep learning and process understanding for data-driven Earth system science. *Nature* **566**, 195–204, 10.1038/s41586-019-0912-1 (2019). 30760912 10.1038/s41586-019-0912-1

[51] Afrasiabian, Y., Contiz, F., Van Cleemput, E., Egerer, M. & Yu, K. Biodiversity monitoring in urban community gardens using proximal sensing and drone remote sensing. *Remote Sens. Appl. Soc. Environ.* **39**, 101685, 10.1016/J.RSASE.2025.101685 (2025).

[52] Wadoux, A. M. J. C., Heuvelink, G. B. M., de Bruin, S. & Brus, D. J. Spatial cross-validation is not the right way to evaluate map accuracy. *Ecol. Modell.* **457**, 109692, 10.1016/J.ECOLMODEL.2021.109692 (2021).

[53] Afrasiabian, Y. *et al*. Urban Garden Ground-Cover UAV RGB Orthomosaic Dataset for Semantic Segmentation. *Zenodo. Version* **2**10.5281/zenodo.18757882 (2026).

[54] Lizarazo, I. & Elsner, P. Fuzzy segmentation for object-based image classification. *Int. J. Remote Sens.* **30**, 1643–1649, 10.1080/01431160802460062 (2009).

[55] Colkesen, I. & Kavzoglu, T. The use of logistic model tree (LMT) for pixel- and object-based classifications using high-resolution WorldView-2 imagery. *Geocarto Int.* **32**, 71–86, 10.1080/10106049.2015.1128486 (2017).

[56] Goodfellow, I., Bengio, Y. & Courville, A. *Deep Learning*. (MIT Press, 2016).

[57] Zhao, Y. *et al*. Land-Unet: A deep learning network for precise segmentation and identification of non-structured land use types in rural areas for green urban space analysis. *Ecol. Inform.* **87**, 103078, 10.1016/J.ECOINF.2025.103078 (2025).

[58] Ronneberger, O., Fischer, P. & Brox, T. U-Net: Convolutional networks for biomedical image segmentation. *In Med. Image Comput. Comput.-Assist. Interv. (MICCAI)* 234–241 10.1007/978-3-319-24574-4_28 (2015).

[59] Chen, L. C., Zhu, Y., Papandreou, G., Schroff, F. & Adam, H. Encoder-decoder with atrous separable convolution for semantic image segmentation. *Lect. Notes Comput. Sci. (including Subser. Lect. Notes Artif. Intell. Lect. Notes Bioinformatics)* **11211 LNCS**, 833–851, 10.1007/978-3-030-01234-2_49 (2018).

[60] Iglovikov, V. & Shvets, A. TernausNet: U-Net with VGG11 Encoder Pre-Trained on ImageNet for Image Segmentation. https://arxiv.org/abs/1801.05746v1 (2018).

[61] Deng, J. *et al*. ImageNet: A Large-Scale Hierarchical Image Database. *2009 IEEE Conf. Comput. Vis. Pattern Recognition, CVPR 2009* 248–255 10.1109/CVPR.2009.5206848 (2009).

[62] Kingma, D. P. & Ba, J. L. Adam: A Method for Stochastic Optimization. *3rd Int. Conf. Learn. Represent. ICLR 2015 - Conf. Track Proc*. https://arxiv.org/abs/1412.6980v9 (2014).

[63] Prechelt, L. Early stopping, but when? 55–69 10.1007/3-540-49430-8_3 (1998).

[64] Zhang, Y., Li, K., Zhang, G., Zhu, Z. & Wang, P. DFA-UNet: Efficient Railroad Image Segmentation. *Appl. Sci*. **13**, 10.3390/APP13010662 (2023).

[65] Dong, R., Pan, X. & Li, F. DenseU-Net-Based Semantic Segmentation of Small Objects in Urban Remote Sensing Images. *IEEE Access* **7**, 65347–65356, 10.1109/ACCESS.2019.2917952 (2019).

[66] Shahinfar, S., Meek, P. & Falzon, G. How many images do I need?” Understanding how sample size per class affects deep learning model performance metrics for balanced designs in autonomous wildlife monitoring. *Ecol. Inform.* **57**, 101085, 10.1016/J.ECOINF.2020.101085 (2020).

[67] Sergeev, A. & Del Balso, M. Horovod: fast and easy distributed deep learning in TensorFlow. https://arxiv.org/pdf/1802.05799 (2018).

[68] Zhu, X. X. *et al*. Deep Learning in Remote Sensing: A Comprehensive Review and List of Resources. *IEEE Geosci. Remote Sens. Mag.* **5**, 8–36, 10.1109/MGRS.2017.2762307 (2017).

