# Supplementary material for: A drone imagery dataset for semantic segmentation of urban garden ground covers in biodiversity studies
Source: Sci Data. 2026 Apr 8;13:590. doi: 10.1038/s41597-026-07152-z (PMC13068989; doi:10.1038/s41597-026-07152-z)
Supplement: Supplementary file 1 — Supplementary information [file 41597_2026_7152_MOESM1_ESM.pdf]

Supplementary information for

A drone imagery dataset for semantic segmentation of urban garden ground covers in biodiversity studies

Yasamin Afrasiabian[1], Chenghao Lu[1,2] , Anirudh Belwalkar[1], Hany Elsharawy[1], Xiaoxin Song[1], Ying Yuan[1], Fei Wu[1], Xiang Su[2], Elisa Van Cleemput[3], Monika Egerer[4], Kang Yu[1,*]

[1] Precision Agriculture Lab, TUM School of Life Sciences, Technical University of Munich, Dürnast 9, 85354 Freising, Germany

[2] Department of Computer Science, University of Helsinki, Helsinki, Finland and Department of

Agricultural Sciences, University of Helsinki, Helsinki, Finland

[3] Leiden University College The Hague, Leiden University, Anna van Buerenplein 301, 2595 DG Den Haag, The Netherlands.

[4] Urban Productive Ecosystems, TUM School of Life Sciences, Technical University of Munich, Hans Carl-von-Carlowitz-Platz 2, 85354 Freising, Germany

Supplementary Note 1: Machine Learning Models based on Traditional learning

Random Forest Classifier

The RF model was implemented using scikit-learn's ensemble module, comprising an ensemble of decision trees trained on train subsets of the data. To optimize hyperparameters, we used RandomizedSearchCV, which randomly selects combinations of hyperparameters from predefined distributions. In this approach, for each configuration, the model was trained on the training subset and evaluated on a dedicated validation subset. A PredefinedSplit was applied, assigning fold indices of -1 to training samples and 0 to validation samples, ensuring that validation performance, based on the original validation set, guided parameter selection without leakage. We searched over a wide range of parameters: the number of trees (n_estimators) was tested at 100, 200, 300, 400, and 500; the maximum depth of the trees (max_depth) was set to 10, 20, and 30; and the minimum number of samples per leaf (min_samples_leaf) was set to 1, 3, and 5. Minimum samples to split (min_samples_split: 2, 5, 10), and feature subsampling (max_features: 'auto', 'sqrt', 0.5). Tuning ran with n_jobs=4 to balance the computational load, selecting optimal parameters based on validation accuracy and kappa. After determining the best hyperparameters, we saved a final RF model and trained it exclusively on the original training set. The selected hyperparameters were n_estimators = 500, max_depth = None, min_samples_leaf = 3, min_samples_split = 2, and max_features = 'sqrt'. Details of the tuning procedure and search space are provided in the Supplementary Information.

XGBoost classifier

XGBoost was selected for its efficacy in handling complex, high-dimensional datasets and multi-class classification tasks. The model operates by iteratively constructing a series of decision trees, each correcting the residuals of the previous trees, optimized via gradient descent on a multinomial logistic loss function. Similar to the RF model, we used RandomizedSearchCV to optimize model performance. The hyperparameter search space included, number of trees (ranging from 100 to 500 in steps of 100), the maximum depth of the trees (3, 6, 9, 12, 15), step size for gradient updates (sampled from [0.01, 0.05, 0.1, 0.2]), fraction of samples used per tree (ranging from 0.6 to 1.0 in steps of 0.1), fraction of features used per tree (ranging from 0.6 to 1.0 in steps of 0.1), minimum loss reduction for splits ([0, 0.1, 0.2, 0.3]), and Minimum sum of instance weights per child node ([1, 3, 5]). Same as RF model, hyperparameters identified by RandomizedSearchCV were used to train the final XGBoost model.

Maximum Likelihood Classifier

Quadratic Discriminant Analysis (QDA), a parametric statistical classifier, was employed as the core model, referred to herein as MLC due to its maximum likelihood estimation basis. QDA assumes that each class follows a multivariate Gaussian distribution, characterized by class-specific mean vectors and covariance matrices. Classification is performed by computing the quadratic discriminant function for each class k:

$$\delta_{k(x)} = -\frac{1}{2}log|\Sigma_k| - \frac{1}{2}(x - \mu_k)^{T\Sigma_k^{\{-1\}(x-\mu_k)}} + log\pi_k \qquad (1)$$

where x is the input feature vector, μ_k and Σ_k are the mean vector and covariance matrix of class k, $|\Sigma_k|$ is the determinant of the covariance matrix, and π_k is the prior probability of class k. The class with the highest $\delta_{k(x)}$ is assigned to the sample.

Hyperparameter optimization was conducted using RandomizedSearchCV, aiming to maximize classification accuracy for the QDA model. The parameter grid encompassed the regularization parameter, which stabilizes covariance matrix inversion by shrinking the covariance matrix toward a diagonal form as $\Sigma'_{\{k\}} = (1 - reg_{param}).\ \Sigma_{\{k\}} + reg_{param} \cdot diag(\Sigma_{\{k\}})$, ranging from 0.0 to 1.0 in 21 evenly spaced steps (0.0, 0.05, ..., 1.0), and the class prior probabilities, tested as either empirical priors derived from training data class frequencies or a uniform array where $\pi_{\{k\}} = {}^{1}/_{n_{classes}} = 0.125$ for all eight classes to address potential class imbalance.

Supplementary Note 2: Machine Learning Models based on deep learning

UNet Model

We employed a UNet convolutional neural network for semantic segmentation [55,56]. The UNet architecture consists of a contracting encoder path that captures context through progressive down-sampling, paired with a symmetric expanding decoder path that enables precise localization via up-sampling and multi-scale feature fusion [57]. In our implementation, the encoder portion of the UNet is a ResNet50 deep residual network that was pre-trained on ImageNet [58,59], enabling transfer learning and improving convergence. The network produces an output segmentation map with 9 channels (one per class, including background). During training, these outputs are interpreted as class logits at each pixel, which are passed into a multi-class cross-entropy loss. At inference time, the softmax of these logits is implicitly taken, and each pixel is assigned the class with the highest probability, yielding a predicted class label map the same size as the input patch. All implementation was done in PyTorch, using the segmentation_models_pytorch library for the UNet model definition.

The network was trained using a supervised learning approach on the prepared training set of image patches. We trained for 25 epochs, using a mini-batch size of 16 patches per iteration. The Adam optimizer [60] was used to update model weights, with dynamic learning rate starting at 1×10^{-5}, using a patience of 5 epochs and a reduction factor of 0.1.This low learning rate was chosen to cautiously fine-tune the pre-trained ResNet50 encoder weights without causing drastic changes early in training. We employed automatic mixed-precision training to accelerate computation and reduce memory usage, leveraging PyTorch's autocast and GradScaler. The loss function was the cross-entropy between predicted and true class labels, computed pixel-wise. To ensure the background class (label 0) did not dominate the loss (since background pixels are numerous but not of primary interest), we set the loss function to ignore index 0. In practice, this means that any pixel with ground-truth label 0 is excluded from the loss calculation, focusing the optimization on the eight foreground classes. During each training iteration, the per-pixel loss was averaged over the pixels in the batch (excluding ignored pixels) to obtain a batch loss, and model parameters were updated to minimize this loss.

After each epoch, the model was evaluated on the validation set to monitor performance. However, rather than simply averaging the per-pixel validation loss for each batch (which can be unstable if different validation images have varying amounts of foreground), we adopted a robust aggregation strategy. We computed the cross-entropy loss on the validation patches with no reduction, yielding a loss value for each pixel. All losses for pixels belonging to the eight classes of interest (i.e. excluding background) across the entire validation set were then summed up and divided by the total number of valid pixels, effectively computing a mean loss over all non-background pixels in the validation set. This approach, aggregating at the pixel level over the whole validation set, ensures that the validation loss reflects overall pixel-wise performance and is not unduly influenced by the content of individual batches. The model's weights after each epoch were saved if the validation loss had improved (decreased) compared to previous epochs. The final model selected for testing was the one with the lowest validation loss observed during the 25 epochs [61].

DeepLabV3+ Model

We adopted the DeepLabV3+ semantic segmentation architecture [62] for this task, configured to output nine classes (eight ground cover classes plus background). DeepLabV3+ is a state-of-the-art fully convolutional network that extends the earlier DeepLab models [63–65] by incorporating an encoder–decoder design [62]. In our implementation, the encoder is a ResNet-50 convolutional neural network [66] pre-trained on ImageNet [59], which serves as a powerful feature extractor. The ResNet-50 backbone (with 50 layers) provides multi-scale feature maps; leveraging pre-trained ImageNet weights initializes the network with rich low-level and high-level feature representations. The DeepLabV3+ decoder includes an Atrous Spatial Pyramid Pooling (ASPP) module to capture multi-scale contextual information, and a lightweight decoder module to refine segmentation outputs, especially along object boundaries. We modified the final classifier layer of the network to produce 9 output channels, corresponding to the 9 target classes including the background.

Both DeepLabV3+ and UNet models were trained and validated under identical conditions: 25 epochs, batch size of 16, dynamic learning rate, and cross-entropy loss excluding background pixels.

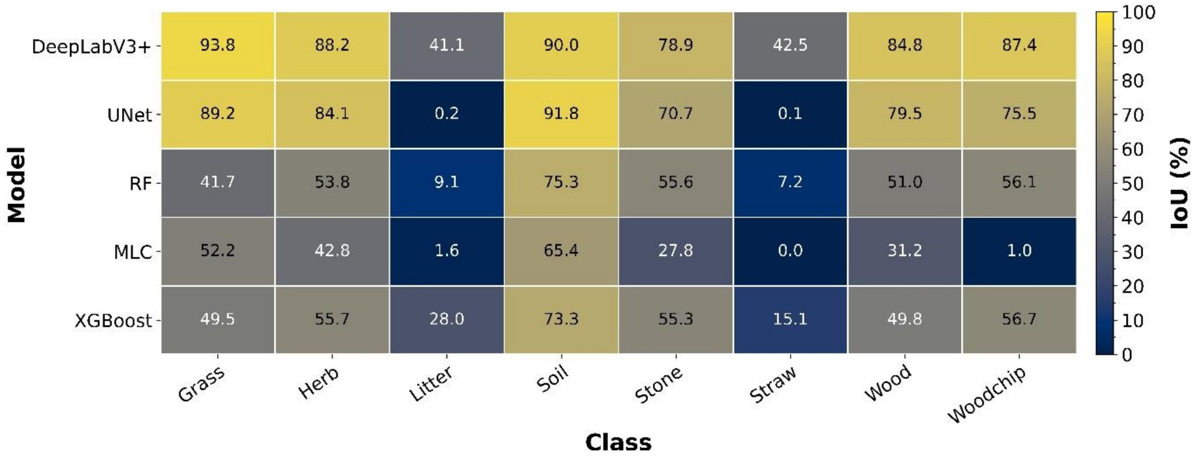

Figure S1. Class-wise Intersection-over-Union (IoU) for semantic segmentation across eight classes (Grass, Herb, Litter, Soil, Stone, Straw, Wood, Woodchip) and five models (DeepLabV3+, UNet, Random Forest (RF), Maximum Likelihood Classifier (MLC), and Extreme Gradient Boosting (XGBoost))

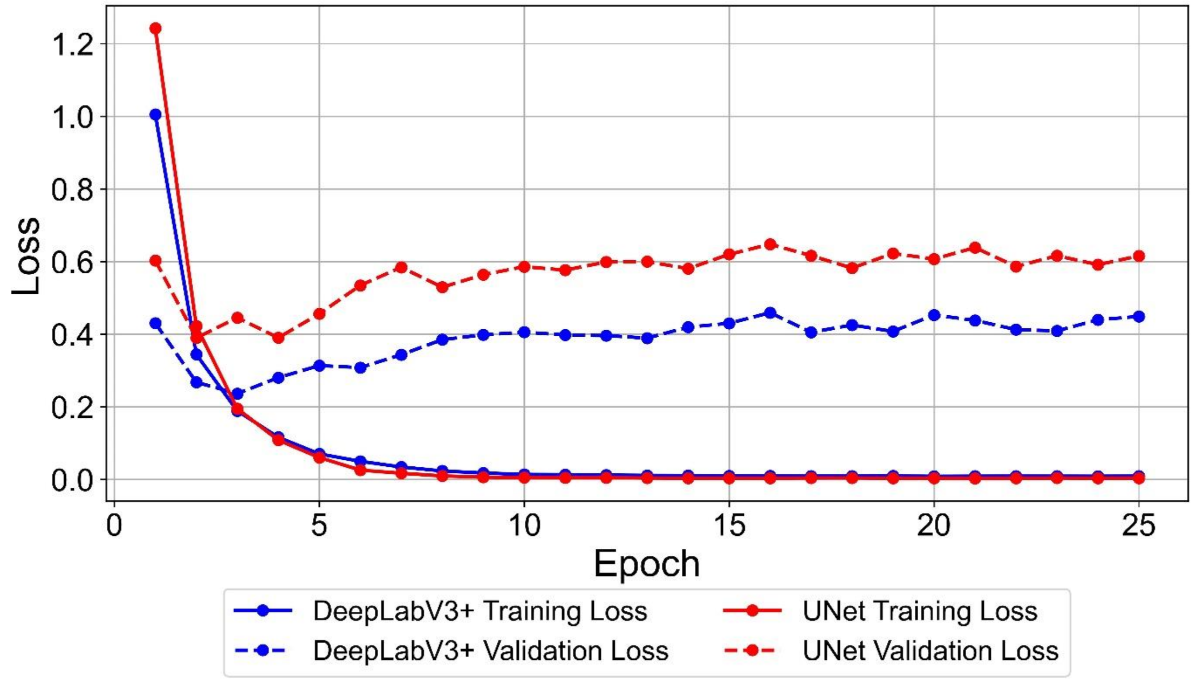

Figure S2. Comparison of the training and validation loss curves for the DeepLabV3+ and UNet models over 25 epochs. The best model is selected at the epoch with minimum validation loss; later epochs can show overfitting.

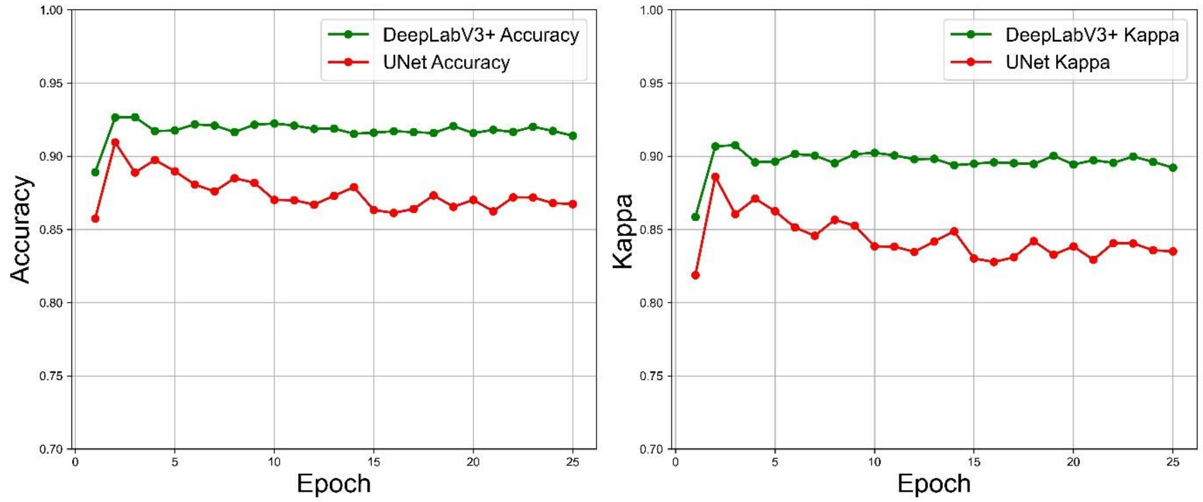

Figure S3. Epoch-wise comparisons of Accuracy (left) and Cohen's Kappa (right) for DeepLabV3+ (green) and UNet (red) over 25 training epochs.
